# Identification of transcriptome-wide, nut weight-associated SNPs in *Castanea crenata*

**DOI:** 10.1038/s41598-019-49618-8

**Published:** 2019-09-11

**Authors:** Min-Jeong Kang, Ah-Young Shin, Younhee Shin, Sang-A Lee, Hyo-Ryeon Lee, Tae-Dong Kim, Mina Choi, Namjin Koo, Yong-Min Kim, Dongsoo Kyeong, Sathiyamoorthy Subramaniyam, Eung-Jun Park

**Affiliations:** 1 Forest Biotechnology Division, National Institute of Forest Science, Suwon, 16631 Republic of Korea; 2 Plant Systems Engineering Research Center, Korea Research Institute of Bioscience and Biotechnology, Daejeon, 34141 Republic of Korea; 3 Research and Development Center, Insillicogen Inc, Yongin, 16954 Republic of Korea; 4 Department of Biological Sciences, Sungkyunkwan University, Suwon, 16419 Republic of Korea; 5 Plant Resources Industry Division, Baekdudaegan National Arboretum, Bonghwa, 36209 Republic of Korea; 6 Korean Bioinformation Center, Korea Research Institute of Bioscience and Biotechnology, Daejeon, 34141 Republic of Korea; 7 Laboratory of Developmental Biology and Genomics, Seoul National University, Seoul, 08826 Republic of Korea

**Keywords:** Genetic markers, Plant breeding

## Abstract

Nut weight is one of the most important traits that can affect a chestnut grower’s returns. Due to the long juvenile phase of chestnut trees, the selection of desired characteristics at early developmental stages represents a major challenge for chestnut breeding. In this study, we identified single nucleotide polymorphisms (SNPs) in transcriptomic regions, which were significantly associated with nut weight in chestnuts (*Castanea crenata*), using a genome-wide association study (GWAS). RNA-sequencing (RNA-seq) data were generated from large and small nut-bearing trees, using an Illumina HiSeq. 2000 system, and 3,271,142 SNPs were identified. A total of 21 putative SNPs were significantly associated with chestnut weight (false discovery rate [FDR] < 10^−5^), based on further analyses. We also applied five machine learning (ML) algorithms, support vector machine (SVM), C5.0, *k*-nearest neighbour (*k*-NN), partial least squares (PLS), and random forest (RF), using the 21 SNPs to predict the nut weights of a second population. The average accuracy of the ML algorithms for the prediction of chestnut weights was greater than 68%. Taken together, we suggest that these SNPs have the potential to be used during marker-assisted selection to facilitate the breeding of large chestnut-bearing varieties.

## Introduction

The chestnut is widely cultivated as a food crop in Asian and European countries, due to its high nutrient contents combined with its low fat content^1,2^. Moreover, it is the only nut known to contain vitamin C, with 40 mg of vitamin C per 100 g of raw product, which represents 65% of the United States Department of Agriculture (USDA) recommended daily uptake of vitamin C (USDA nutrient database, http://ndb.nal.usda.gov). Moreover, *Castanea* trees contribute to the maintenance of the ecosystem and soil fertility^3,4^.

Since 1997, global chestnut production has increased sharply, reaching its maximum level (approximately 2 million tons) in 2009^5^. According to a report published by the Food and Agriculture Organization (FAO) in the United States, chestnut production has steadily increased in Asia through 2014, and chestnuts grown in Asia accounted for 89.6% (1.8 million tons) of the world chestnut production that year^5^. The Republic of Korea is the second largest chestnut producer^5^. According to the Korean export index, chestnuts represent the largest exported forest crop for Korea, and chestnut exports increased by approximately 23% in 2017 compared with 2016^6^. In Korea, the cultivar Arima (*C*. *crenata*) and its hybrids, including *C*. *crenata* × *C*. *mollissima*, are widely cultivated in the fields^7^. Moreover, new Korean indigenous cultivars, such as Mipung^8^ and Jahong^9^, have been developed to improve production under various environmental conditions. The nut weight represents one of the most important traits that can determine the economic success of a grower^1^.

Conventional tree breeding (CTB) involves the planned interbreeding between closely related individuals to produce new cultivars that express desirable traits, such as enhanced productivity or being easier to harvest. Using CTB, the development of new cultivars that express beneficial traits can take a long time, making it difficult to meet the grower’s demands. Moreover, the long life cycle of trees, including chestnut trees, becomes a barrier to CTB compared with the breeding of annual crops. Recently, molecular markers have been identified and used for marker-assisted selection^10^. Representative molecular markers include restriction fragment length polymorphisms, randomly amplified polymorphic DNA, amplified fragment length polymorphisms, simple sequence repeats, inter-simple sequence repeats, and single nucleotide polymorphisms (SNPs)^11,12^. In general, most commercially desirable traits are quantitative traits associated with large numbers of genes. The rapid growth in next-generation sequencing technologies (NGS), combined with various statistical methods, has facilitated the use of genome-wide association studies (GWASs)^13^. In particular, genome-based breeding reduces the time required for various breeding schemes^14,15^. Furthermore, a transcriptome-based GWAS, which is a type of GWAS, uses transcriptome sequencing data to identify variants within the coding regions of the genome^16^. Transcriptome-based GWAS has several advantages compared with the traditional GWAS. First, transcriptome analysis can enhance the power of verifying associated genes by overcoming the potential allelic heterogeneity in SNP-based GWASs^17^, when a number of alleles act through one gene to influence a trait^18^. Second, the transcriptome-based method can reduce the interference from population heterogeneity by various arrangements of tagging different SNPs to the same causal variants^19^. Third, transcriptome-based analysis also practically reduces the burden of multiple testing for traditional GWASs. Furthermore, the gene is the functional unit in the genome with high consistency across populations, which is the major target used by most of the subsequent bioinformatics analyses^16^. Machine learning (ML) is the study of algorithms and statistical models that facilitate software applications to increase the accuracy of outcome predictions without explicit instructions. To predict phenotypes, large-scale genotypic information across the whole genome has been subjected to ML approaches. ML is the most effective method for predicting phenotypes based on genotypes and has been widely applied in various population studies^20,21^. For example, an ML model was constructed to predict rust resistance in wheat from a genomic selection^22^, and have also been applied to other genotype classifications^23^. Therefore, transcriptome-based GWAS combined with ML analysis might be one of the effective and operational methods to predict the phenotype, which is the result of interactions between the organism’s complement of genes.

Our study aimed to predict the transcriptome-wide SNPs that are closely associated with nut weights. Five ML algorithms, including random forest (RF), support vector machines (SVM), *k*-nearest neighbour (*k-*NN), partial least squares (PLS), and C5.0^24^, were used to predict nut weights with 21 SNPs. Through our association study and the ML approaches, we identified 21 SNPs associated with nut weights that were able to clearly discriminate between large and small nut-bearing populations. Hence, we suggest that the ML approach is an effective method for the prediction of nut weights. This study represents the first attempt to identify highly significant SNPs associated with nut weights in Korean chestnut trees.

## Results

### RNA sequencing and variant calling

**Figure 1:**
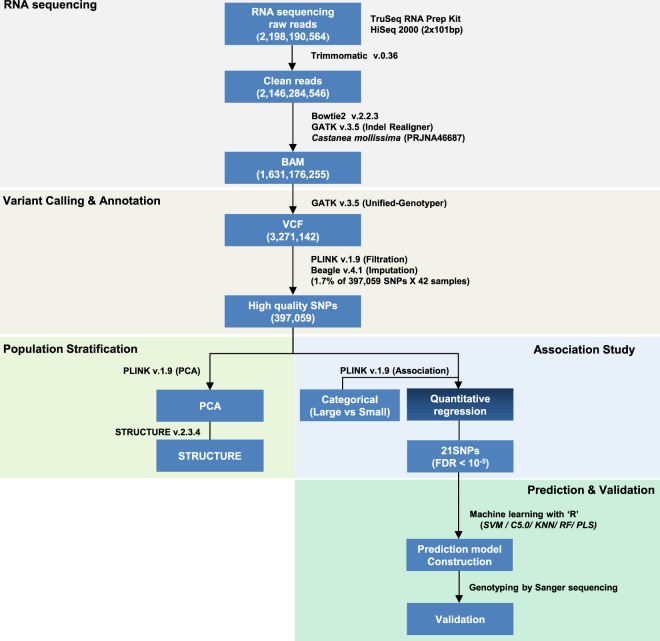
Overview of the systematic SNP analysis. A total of 21 SNPs (FDR < 10^−5^) related to chestnut weight were obtained and validated, as shown above. Individual cut-off values and bioinformatic programs are denoted parallel to each step. Illumina RNA sequencing was performed using 42 chestnut trees, categorised as either large or small nut-bearing populations. For the machine learning algorithms, 70% of the 42 chestnut tree genotypes were randomly used for training and the remaining 30% were used for testing. For further validation, using Sanger sequencing, another 46 chestnut tree genotypes were used as the validation set.

To obtain variations in transcribed sequences, 42 chestnut accessions were selected for the training set, according to their nut weights (Supplementary Table S1). The training set was divided into large (>25 g/nut) and small (<15 g/nut) nut-bearing groups (Supplementary Table S1), whereas the nut weights in the validation sets (n = 46) ranged from 7.5 g to 25.0 g (Supplementary Table S2). Total RNAs were extracted and sequenced using an HiSeq. 2000 platform (Illumina, San Diego, CA, USA). An average of 52.3 million raw reads were generated from the training set. After quality control and trimming processes were applied, an average of 51.1 million high-quality, clean reads were obtained (Fig. 1, Supplementary Table S3). Clean reads were subsequently mapped to the Chinese chestnut (*Castanea mollissima*) genome, and the resulting mapping rate was 84.4%, on average (Supplementary Table S3).

High-quality, clean reads were further processed according to the systematic variant calling protocol (Fig. 1). A total of 3,271,142 SNPs were generated, using the gene analysis tool kit (GATK) pipeline, and annotated, using SnpEff v.4.2. Of these, 397,059 SNPs remained after the data were filtered using cut-off values, including genotype rate ≥90%, minor allele frequency ≥5%, and Hardy-Weinberg equilibrium value < 0.001. These high-quality SNPs were then separated by genomic position (Supplementary Table S4).

### Genetic diversity and population structure analysis

**Figure 2:**
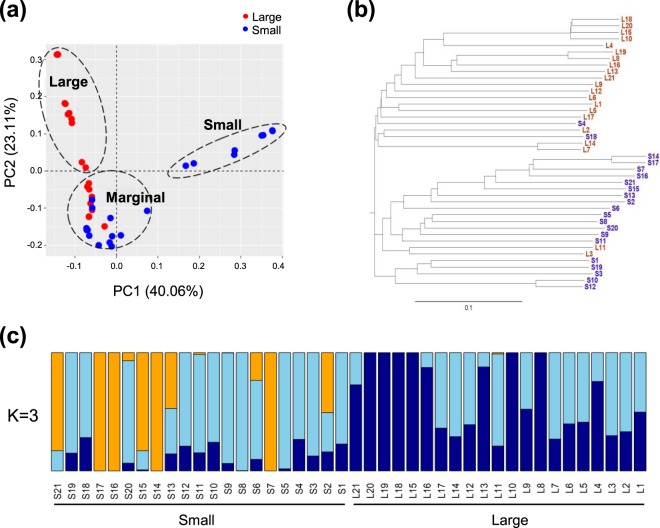
Genetic structures of 42 individual chestnut trees. (**a**) Plots of the first principle components, using 397,059 filtered SNPs. Individual accessions are shown as coloured dots that represent either large (red) or small (blue) nut-bearing populations. The percentage of variance is described in the axis. (**b**) Neighbour-joining phylogenetic tree of 42 chestnut accessions, based on SNPs. The subclades represent either a large (red) or small (blue) nut-bearing population, and the scale bar unit (0.1) is displayed below the tree. (**c**) Population structure histogram inferred using the Bayesian model-based STRUCTURE clustering method. The colour of each bar represents a specific cluster of large and small nut-bearing populations at an optimal value of K = 3.

Genetic diversity within the training set was assessed using the 397,059 filtered SNPs. The principle component analysis (PCA) indicated that the 42 individual chestnut trees could be classified into three clusters, large (n = 12), small (n = 8), and marginal (n = 22), using the first and second principal components, PC1 and PC2, respectively (Fig. 2a). Phylogenetic tree analysis, using the neighbour-joining method, also returned large, small, and partially coexistent groups (Fig. 2b). Several trees (S4, S18, L3, and L11) were included in the marginal subset, as shown by the PCA analysis. To estimate the population structure, a STRUCTURE analysis, based on the Bayesian model-based clustering method, was performed. The optimal number of groups was three, based on the maximum likelihood and ΔK value (K = 3) (Fig. 2c). In this model, the large and small populations consisted of two and three distinct sub-populations, respectively, suggesting that the small population experienced more genetic drift than the large population.

### SNPs associated with nut weight phenotypes

**Figure 3:**
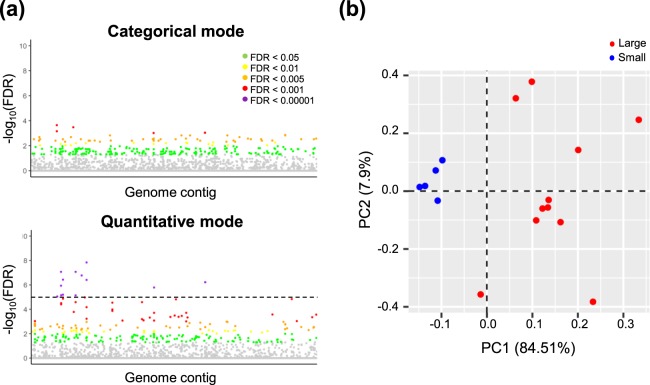
Clustering of SNPs associated with nut weight phenotypes. (**a**) GWAS-based Manhattan plots to illustrate the significant FDR levels for the SNPs associated with nut weight. The *x*-axis represents the relative density of SNPs mapped to the genome contig. The *y*-axis represents the -log 10 FDR level of the SNPs. SNPs at different FDR are represented by different colours: green (0.05), yellow (0.01), orange (0.005), red (0.001) and violet (0.00001). (**b**) Principal component analysis (PCA) showing 21 SNPs with FDR levels < 10^−5^ in the large (red) and small (blue) nut-bearing populations. The percentage of variance is described in the axis.

A GWAS was performed to explore the association between nut weights and SNPs (Fig. 3). Categorical and regression association studies (false discovery rate [FDR] < 0.05) respectively identified 365 and 341 SNPs. Among them, 192 SNPs were identified in both association studies (Supplementary Fig. 1). Consequently, a total of 514 SNPs were selected and mapped to the genomic contigs of Chinese chestnut (Fig. 3a). Finally, we identified 21 SNPs showing strong genetic correlations with nut weight (FDR < 10^−5^, Table 1), by the quantitative regression. PCA analyses using the 21 SNPs showed clear groupings between small and large populations (Fig. 3b). These 21 SNPs were further used for the ML approach and validation.

**Table 1:** List of 21 SNPs with strong genetic correlations with chestnut weight (FDR < 10^−5^).

Contig ID (Scaffold No: SNP Location)	Minor allele	Major allele	Variant Type	FDR	Gene name	Function
scaffold01190:13428	T	G	Nonsynonymous	1.46E-08	Pentatricopeptide repeat-containing protein	Seed development^31–35^
scaffold01019:66350	G	A	Nonsynonymous	1.70E-07	E3 ubiquitin ligase	ABA Signaling^36–39^
scaffold01019:66232	A	G	Nonsynonymous	1.70E-07		
scaffold00551:8613	A	G	Nonsynonymous	6.32E-06	ABC1 protein	Seed development^40^
scaffold00859:85468	T	C	Nonsynonymous	8.62E-08	Anaphase-promoting complex subunit 1	Cell cycle control^55^
scaffold00485:2380	T	C	Nonsynonymous	8.62E-08	1-aminocyclopropane-1-carboxylate oxidase	Fiber cell elongation^56^
scaffold00406:56142	T	C	Nonsynonymous	8.98E-06	Zinc metalloprotease	Proteolysis^57^
scaffold00485:53410	C	T	Nonsynonymous	1.16E-06	Omega-3 fatty acid desaturase	Abiotic stress^58,59^
scaffold01190:53971	A	G	Nonsynonymous	3.90E-07	SER/THR-protein kinase-like protein	Seed oil accumulation^60^
scaffold00485:18530	G	A	Synonymous	8.62E-08	Anaphase-promoting complex subunit 1	Cell cycle control^55^
scaffold00491:55296	A	T	Synonymous	6.99E-06	LOB domain-containing protein	Lateral organ formation^61,62^
scaffold00859:92916	T	A	Synonymous	7.21E-06	Anaphase-promoting complex subunit 1	Cell cycle control^55^
scaffold01019:66111	G	A	Synonymous	1.70E-07	E3 ubiquitin ligase	ABA Signaling^36–39^
scaffold01019:57473	A	G	Synonymous	1.70E-07		
scaffold01019:57173	C	T	Synonymous	1.70E-07		
scaffold01190:53970	A	G	Synonymous	3.90E-07	SER/THR-protein kinase-like protein	Seed oil accumulation^60^
scaffold00485:49474	C	T	3’ UTR	1.16E-06	N-(5’-phosphoribosyl) anthranilate isomerase 1	Leaf development^63^
scaffold00551:98229	T	C	Downstream	3.74E-07	DNA replication complex GINS protein	DNA replication^64^
scaffold00551:85919	G	A	Downstream	3.74E-07	EARLY FLOWERING 4	Flowering^65^
scaffold00406:46909	T	C	Nonsynonymous	8.98E-06	Unknown	N.A.
scaffold00406:46801	C	G	Nonsynonymous	8.98E-06	Unknown	N.A.

### Evaluation of nut weight predictions in a training population

**Figure 4:**
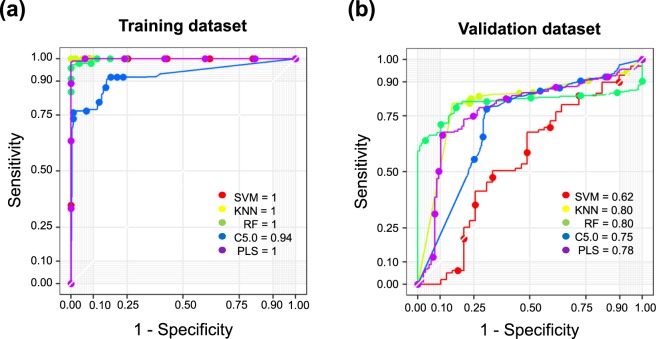
Machine learning algorithm for nut weight prediction accuracy. Receiver operating characteristic (ROC) curves for 21 SNPs (FDR < 10-5) using five machine learning algorithms: support vector machines (SVM), *k*-nearest neighbour (*k-*NN), random forest (RF), C5.0 and partial least squares (PLS). Each area under the curve (AUC) shows the average of 10 cross validations for (**a**) the training dataset (n = 42) and (**b**) the validation dataset (n = 46).

To investigate the predictive potential of 21 putative SNP markers for nut weight, we first divided the entire dataset (n = 42) into a training dataset (29 cases) and a test dataset (13 cases) and then evaluated the model using five ML algorithms, including SVM, PLS, RF, *k-*NN and C5.0. The resulting prediction accuracy was 98.7%, on average (Fig. 4a, Supplementary Table S5), indicating that the ML-based prediction models performed well for the training population.

A new chestnut population (n = 46) was assessed to validate the performance of the ML models during the prediction of nut weights. The average prediction accuracy of the five ML models was 74.9% for the validation population (Fig. 4b, Supplementary Table S5). Among the five ML algorithms, both *k-*NN and RF outperformed the other algorithms for both the training and validation populations. The values of each area under the curve (AUC) from the quantitative regression mode in the association study were calculated for the 21 SNPs (FDR < 10^−5^). Overall, the results demonstrated that the ML-based predictive models performed well in the training dataset (Fig. 4a). In the validation dataset, the ML algorithms performed with greater than 75% accuracy, except for the SVM model (Fig. 4b). The RF algorithm outperformed the other algorithms for both the training and validation datasets. Similarly, the use of different sets (A to H) of SNPs as predictive variables was evaluated in the training population (Supplementary Table S6). These results indicated that the RF algorithm provided the best accuracy (as measured by the mean receiver operating characteristic [ROC]) for all SNP sets compared with the other algorithms.

### Genotyping and validation of the SNPs among the validation population

Even though the 21 SNPs (FDR < 10^−5^) were validated by the ML application, we re-evaluated these 21 SNPs and then performed genotyping PCR using a different population (n = 46, validation dataset). To evaluate the 21 SNPs in the validation dataset, the flanking sequences of these SNPs were genotyped using Sanger sequencing and specific primers (Supplementary Tables S7 and S8). Remarkably, SNP 13428 caused a missense mutation by replacing a guanine nucleotide (G) with a thymine (T), resulting in a shift from a small nut-bearing population to a large nut-bearing population (Table 1, Supplementary Table S8). This SNP is located in the well-studied *pentatricopeptide repeat* (*PPR*) gene, which plays an important role in determining the nut weights of other plants. The rate of the polymorphic status of this locus across 21 representative individuals primarily depended on the frequency of the minor allele in the large nut-bearing population (Supplementary Table S8). Moreover, the minor alleles of five SNPs (57173, 57473, 66111, 66232, and 66350), located in scaffold 01019, which encodes an E3 ubiquitin ligase component protein, were frequently detected in large nut-bearing groups (Table 1, Supplementary Table S8). In addition, the minor alleles of three SNPs (2380, 85468, and 92916) in the *Anaphase-promoting complex* (*APC*) gene were primarily detected in the large nut-bearing groups (Table 1, Supplementary Table S8). These results suggest that the sequences involved in seed development are diverse among the large nut-bearing groups, and that attempts to improve the quality of the nuts likely resulted in the artificial selection of these SNPs.

**Figure 5:**
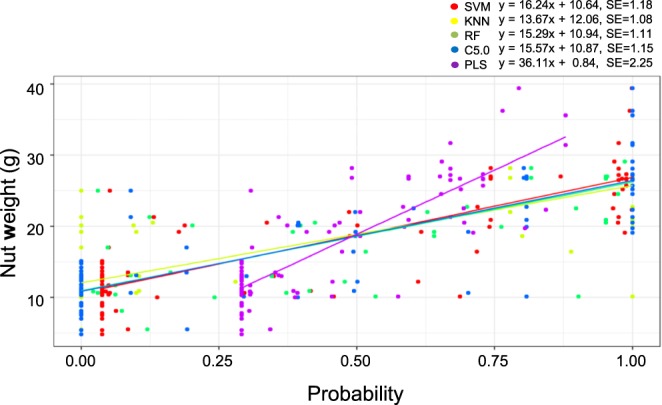
Scatter plot showing the prediction accuracy of nut weights. Lines represent the regressions of the expected nut weights (n = 88). The coefficients of determination (R^2^) for the five ML algorithms (plots in multiple colours) were calculated as the proportion of variability between the regression graph and the observed nut weights. R^2^ values for each algorithm: SVM = 0.68, *k-*NN = 0.65, RF = 0.68, C5.0 = 0.68, and PLS = 0.70. SE represents the standard error of the nut weight estimate.

Similarly, regression modes were constructed for both the training and validation populations (n = 88), using the same ML algorithms to predict nut weights according to genotypes. The overall performance yielded an average accuracy of 68% (*R*^2^ value) (Fig. 5). For this analysis, the PLS algorithm outperformed the other algorithms. Thus, we obtained a mathematical structure for calculating nut weight according to genotype, using the regression mode. In addition, we evaluated the 21 SNPs identified as being important for prediction accuracy and compared the predicted nut weights with the actual nut weights in the validation population (n = 46) (Supplementary Table S8). The predicted probability of a given nut weight was highly correlated with the actual nut weight in the 46 validation samples. Therefore, our data suggested that the transcriptome-based GWAS enabled the identification of highly related SNPs associated with nut weights and that ML model-based prediction might represent a useful approach for discriminating nut weights from chestnut datasets.

## Discussion

Many breeders have evaluated genetic and environmental variations to improve breeding efficiency^25^. Desirable trait-based selective breeding is also an important process. Phenotypic and environmental factors, including plant growth and disease resistance, can be assessed at an early stage, such as during seed germination. However, traits related to yield are highly dependent on the plant life cycle. Perennial trees tend to remain in a juvenile phase for longer than other plants, requiring space and effort to maintain the tree until it enters the reproductive phase^25^. Therefore, improving the evaluation accuracy is more important than increasing the number of trees^26^. A transcriptome-based SNP approach can efficiently address these problems. Additionally, transcriptome-based GWAS represents a cost-effective and time-saving method for developing large-scale markers^16^, especially compared with the general GWAS or other conventional marker identification assays. Recently, transcriptome-based GWAS has been successfully applied to human diseases^16,17^.

The transcriptome-based SNP approach can be applied to non-model species without a reference genome^27^. Although the whole genome sequencing results for Chinese chestnut (*C*. *mollissima*) have recently been released, it is difficult to apply these data to Korean naturalised chestnuts, due to sequence variations within species^28,29^. Thus, we first conducted the transcriptome sequencing analyses on chestnut trees located in various areas of South Korea and reported the transcriptome-wide polymorphisms found in the coding regions associated with nut weight. An average of 84.4% of the coding genes found in *C*. *crenata* could be mapped onto *C*. *mollisima*, an average of 83.1% of which was uniquely mapped (Supplementary Table S3). Despite sequencing for sufficient matches between the identified SNPs and known chestnut sequences, the establishment of a standard reference genome for the Korean chestnut is necessary for accuracy and for comprehensive sequencing analyses. The establishment of a standard genome would facilitate the study of genetic variations among chestnuts, through linkage mapping between Korea, China and Japan chestnuts^29,30^.

Our study is the first study to utilise SNP identification to determine the weights of the chestnuts grown by Korean chestnut trees. We systemically predicted 21 SNPs among 14 genes, which correlated strongly with nut weights in Korean chestnuts (Table 1). In total, 11 nonsynonymous variations were identified and 9 of 11 nonsynonymous variations were annotated in eight genes identified their function. These nine nonsynonymous variations might affect gene functions and led phenotypic differences of chestnut. Among the identified genes were genes encoding PPR proteins, a large family of proteins that are extensively involved in various plant physiological process, especially seed development^31^. The loss-of-function *EMP8* mutant showed severely arrested embryonic and endosperm development, and the *EMP9* mutation delayed embryogenesis and plant growth through mitochondrial RNA editing in maize. Thus, *PPR*, *EMP8* and *EMP9* are involved in the development of seeds in maize^32,33^. Similarly, the DYW-type PPR protein regulates RNA editing and is essential for early seed development in *Arabidopsis thaliana*^34^. *Small kernel 1* encodes a PPR protein that is required for seed development in maize and rice^35^. In addition, the other highly active genes listed in Table 1 are E3-ubiquitin-protein ligases, which have been widely studied for their roles in the regulation of abscisic acid (ABA) signalling, seed germination, and growth processes in plants^36^. For example, plants with mutations in E3 ubiquitin-protein ligases associated with ABA signalling have been adapted to various environmental factors to regulate seed size^37–39^. In the case of APC gene, two additional synonymous variations were also identified. APC can also affect the fruit size by regulating cell cycle regulation in large fruits. Similarly, *Arabidopsis* ATP-binding cassette (ABC) transporters have been reported to regulate seed size through lipid accumulation during development^40^. In addition, DNA replication complex GINS and early flowering 4 (ELF4) genes are also involved in cell cycle regulation and flowering, respectively. These genes also may affect fruit size in large fruits. With this evidence, we suggest that the 21 SNPs identified in this study across 14 genes are highly likely to be associated with nut weight. Thus, functional biological studies of these 14 genes will provide a better understanding of the mechanisms of action underlying nut weight.

To validate these markers, we evaluated 21 SNPs related to nut weights using five ML algorithms. Using these SNPs as markers during supervised ML applications, most of the prediction models (4 out of 5) predicted nut weights with high accuracy, both during training and during external validation (Fig. 4 and Supplementary Table S6). These ML algorithms were capable of determining 68% of the nut weights according to genotype, in both the training and validation datasets (Fig. 5). However, SNP identification using RNA-seq has some limitations. SNPs associated with other genomic structural elements were overlooked in favour of those associated with nut weight. Moreover, the samples used in this study were randomly collected, rather than through the use of a controlled breeding scheme; therefore, obtaining an exact heterogeneous SNP pattern was not possible. Our Sanger sequencing results demonstrated the accuracy of SNP validation. For this experiment, 46 chestnut trees were randomly selected, and the 21 SNPs were examined in this population using Sanger sequencing. Of the 21 SNPs, 15 SNPs were found to be highly reliable; however, 6 SNPs showed low genotype efficiency among the 46 chestnut trees in the validation dataset (Supplementary Table S8). This result indicates that further external testing groups are necessary to improve the accuracy of SNP validation. An additional filtering step to identify putative SNPs at each locus may be required.

Overall, the results suggest that these SNPs represent a valuable resource for chestnut breeding, especially for nut weight traits. Our research will shorten the time required for chestnut breeding and can be used to improve nut weights, as desired by growers.

## Materials and Methods

### Plant materials

Samples were collected from Korean chestnut trees in the experimental forest of Hwaseong-si, Gyeonggi-do, Republic of Korea. The nuts from each tree were collected at stage II, 14 days after pollination, and the samples were frozen in liquid nitrogen and stored at −80 °C following RNA extraction.

### Illumina RNA library preparation and sequencing

To obtain high-throughput transcriptome data from Korean chestnuts, we implemented Illumina-based NGS sequencing. Total RNA was extracted from individual samples, using TRIzol reagent (Invitrogen), according to the manufacture’s protocol. Total RNA was then quantified using a Nano drop spectrophotometer (Thermo Scientific), and the quality was assessed using an RNA 6000 Nano assay kit (Agilent) and a Bioanalyzer 2100 (Agilent). NGS sequencing libraries were generated from 1 µg of total RNA, using the TruSeq RNA Sample Prep kit (Illumina) according to the manufacture’s protocol. In brief, the poly-A containing RNA molecules were purified using a poly-T oligo attached to magnetic beads. After purification, the total poly-A RNA was fragmented into small pieces, using divalent cations under elevated temperatures. The cleaved mRNA fragments were reverse transcribed into first strand cDNA, using random primers. Short fragments were purified using a QiaQuick PCR extraction kit and resolved with elution buffer for the end reparation and the addition of poly-A. Subsequently, the short fragments were connected with sequencing adapters. Each library was separated by an adjoining and distinct molecular identifier tag. The resulting cDNA libraries were then paired-end sequenced (2 × 101 bp) for use as individual samples with the HiSeq 2000 system (Illumina).

### Variant calling from RNA-seq data

The complete workflow of this study is delineated in Fig. 1. The sequence reads were checked for contaminated adapters and low-quality reads, using Trimmomatic v.0.36^41^, and mapped to the reference genome of Chinese chestnut (*Castanea mollissima*, version 1.1) from Hardwood Genomics Project (https://hardwoodgenomics.org)^42^, using bowtie2 v.2.2.3^43^. To perform filtering on read lengths, reads with average quality scores that were too high (N nucleotide ratio higher than 10%) or too low (less than Q20) were removed. The scaffold genome sequence is publicly available in the NCBI GenBank repository (accession number: KN214215-KN234744, and BioProject accession: PRJNA46687 at http://www.ncbi.nlm.nih.gov/genbank). To optimise the small insertion and deletion artefacts, the reads were re-mapped to the reference with IndelRealigner, and the base-pair quality scores (QUAL) were calibrated using count covariates and the table recalibration functions in GATK v.3.5^44^, as instructed in the best practices protocol. The variants for individual samples were called by UnifiedGenotyper, using the variant call format (GVCF) and the filters normalised quality score (NQS) ≥ 2 and mapping quality (MQ) ≥ 40 to obtain high-quality SNPs. Finally, the SNPs were annotated using SnpEff v.4.2^45^, and the missing genotypes were imputed by Beagle v.4.1^46^ based on the linkage dis-equilibrium (LD) score.

### Dataset for the training population

In the training population, we used 42 specimens that were categorised into two groups (large and small) according to their nut weights (Supplementary Table S1). The training population was sequenced using the RNA-seq protocol. For the large group, the nut weights ranged from 25.5 g to 39.4 g, whereas in the small group, the nut weights range from 4.8 g to 14.2 g (Supplementary Table S1). Further, the large group was treated as the experimental group and the small group was treated as the control group for all subsequent statistical analyses.

### Validation population

For further validation, 46 chestnut trees were used as the test population (Supplementary Table S2), and the SNPs were validated in this population using Sanger sequencing. The 21 significant SNPs that were predicted from training population were amplified using specific primers (Supplementary Table S7). The primers were designed using primer 3^47^ and contained 20 bases from the regions flanking the SNPs (Supplementary Table S7). The size of each amplified PCR product was approximately 290 bp. This dataset was used to evaluate the prediction accuracy of the ML models and to derive the formulas for estimating nut weight according to genotype.

### The genotyping of the validation data

Total genomic DNA was isolated from 46 cultivars of chestnuts using a DNeasy Plant Mini kit (Qiagen, Germany) and the cetyltrimethylammonium bromide (CTAB) method, with minor modifications. PCR amplification was performed in a 20 µL volume using Taq DNA polymerase (RBC bioscience, Taiwan). The genotyping PCRs for this experiment used individual SNP primers, listed in Supplementary Table S7. PCR conditions were as follows: 94 °C for 3 min; 40 cycles at 94 °C for 30 s, 55 °C for 30 s, and 72 °C for 30 s; and extension at 72 °C for 5 min (BIO-RAD T100, USA). The PCR products were sequenced by the Sanger method from forward or reverse primers. The sequences were aligned with their respective chromatograms using the BioEdit software v.7.2.5^48^.

### SNP selection and population stratification

To understand the population structure in a given dataset, the SNPs yielded from the variant calls were subjected to population stratification analysis using PLINK v.1.9^49^ in the PCA mode. The SNPs calculated using this method were further filtered to reduce the false positive predictions using the following filters: genotyping rate ≥ 90%, mapping quality ≥ 40, minor allele frequency (MAF) > 5% and Hardly-Weinberg equilibrium (HWE) < 0.001. Furthermore, the sub-populations (K) of the given samples were estimated through the number of clusters (K), which was obtained using STRUCTURE v.2.3.4^50^, without any prior population information. The ad-hoc static *ΔK* (rate of change in the log probability of data between successive K values) values were used to determine the uppermost hierarchical levels of the population structures present in a given population^51^. The range of clusters (K) was pre-defined from one to seven. The analysis was performed with 20 replicated runs, using 100,000 iterations after a burn-in period of 50,000 runs. The output of the STRUCTURE analysis was visualised using CLUMPAK^52^.

### Estimation of associations between genotype and phenotype

A GWAS was conducted using two modes, categorical association (case vs. control) and quantitative linear regression. The associations between genotype and phenotype for the two modes were calculated using PLINK v.1.9 in the association mode (Fig. 1). The significant SNPs were selected by applying the cut-off of *p*** < **0.01.

### Machine learning prediction and evaluation

Supervised ML was used to construct models to attain greater predictive power from the high-dimensional datasets. Here, we constructed models using significant SNPs (as features), which were selected from the transcriptome-based GWAS. The models were constructed from five learning models: SVM, *k*-NN, RF, PLS, and C5.0^21^. The training population (n = 42) was divided into a training dataset and a validation dataset at a 7:3 ratio for the prediction models. The best model was selected automatically by the caret R package^53^.

To compare the prediction methods, we determined sensitivity, specificity, and accuracy, using the following equations: Sensitivity = [TP/(TP + FN)]; Specificity = [TN/(TN + FP)]; and Accuracy = [(TP + TN)/ (TP + FP + TN + FN)]; where TP was the number of true positives, TN was the number of true negatives, FP was the number of false positives, and FN was the number of false negatives. The performances of the prediction models were assessed using ROC curves, plotting the sensitivity as a function of (1-specificity) for different decision thresholds. Further, to quantitatively compare the ROC curves, we computed the AUC, and significant differences between two ROCs were assessed using a two-tailed Student’s t-test. These evaluation metrics were calculated as explained by Manavalan *et al*.^54^. To calculate the ROC and the AUC, we used the plotROC R package^55^.

## Supplementary information

Supplementary Information

## Data Availability

RNA-seq data were deposited in the Sequence Read Archive database of the National Center for Biotechnology Information under accession number PRJNA497951.
