# Supplementary material for: Identification of transcriptome-wide, nut weight-associated SNPs in Castanea crenata
Source: Sci Rep. 2019 Sep 11;9:13161. doi: 10.1038/s41598-019-49618-8 (PMC6739505; doi:10.1038/s41598-019-49618-8)
Supplement: Supplementary file 1 — Supplementary Information [file 41598_2019_49618_MOESM1_ESM.pdf]

SUPPLEMENTARY INFORMATION

Identification of transcriptome-wide, nut weight-associated SNPs in *Castanea crenata*

Min-Jeong Kang[1†], Ah-Young Shin[2†], Younhee Shin[3,4 †], Sang-A Lee[1], Hyo-Ryeon Lee[1], Tae-Dong Kim[1], Mina Choi[5], Namjin Koo[6], Yong-Min Kim[6], Dongsoo Kyeong[3,7], Sathiyamoorthy Subramaniyam[3] and Eung-Jun Park[1]*

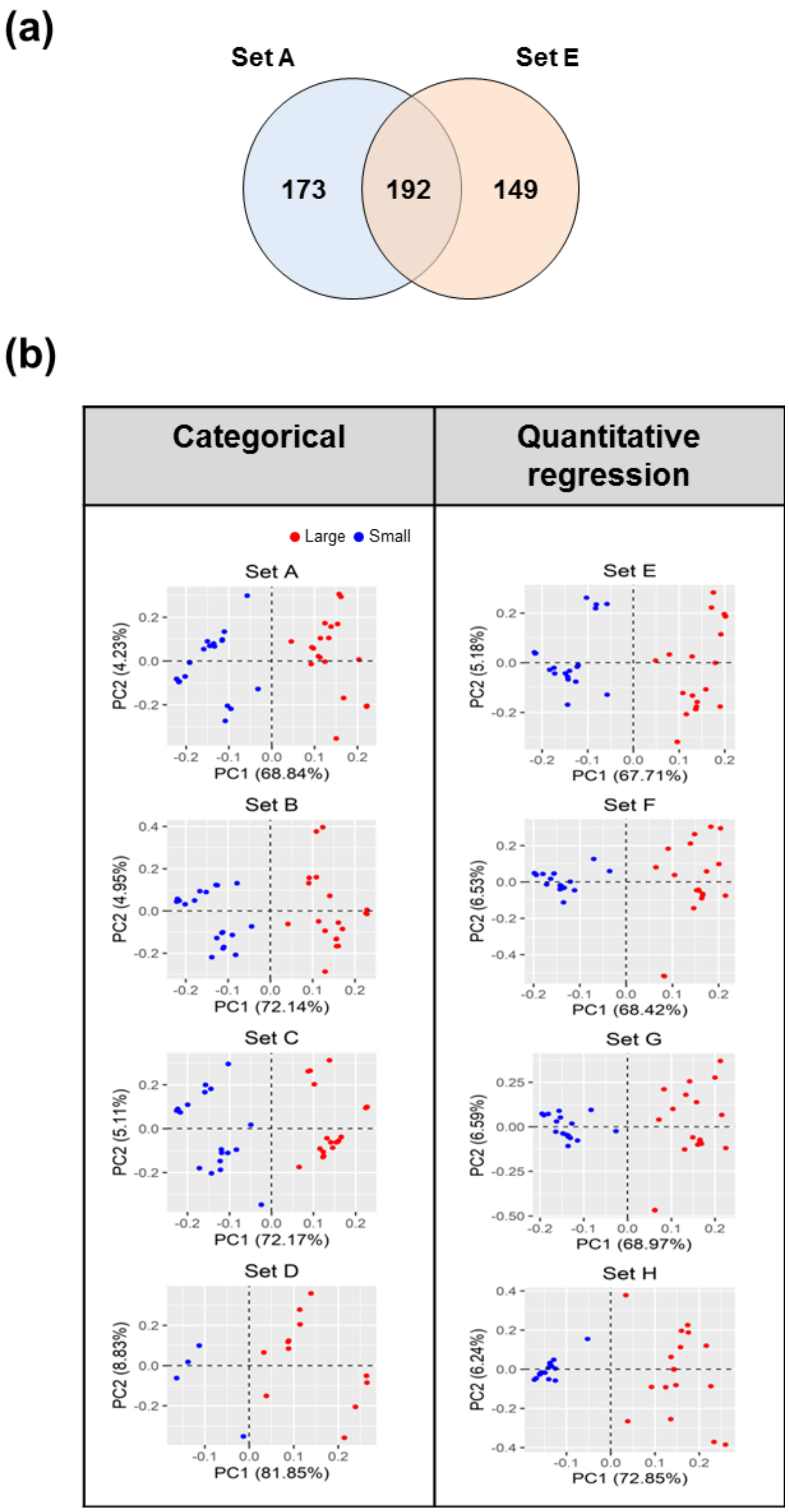

Supplementary Figure S1. SNP selection and clustering by either categorical or quantitative regression mode. (**a**) Venn diagram shows the number of SNPs in categorical data and quantitative data with FDR < 0.05. (**b**) Principal component analysis (PCA) showing important SNPs in large (red) and small population (blue). SNPs with different FDR levels (FDR < 0.05, 0.01, 0.005 and 0.001) are divided into eight sets of subgroups. Set A to D are classified as the categorical mode and Set E to H are categorized by quantitative regression mode.

Supplementary Table S1. List of chestnut accessions for the training sets (n=42).

No.	Name	Nut weight (g/FW)
L1	Chukwang	39.4 ± 3.1
L2	Amsu 2	36.2 ± 2.7
L3	Jangpyung	35.6 ± 4.2
L4	GwangyangB(GY-2)	31.7 ± 3.8
L5	Sangpi	31.4 ± 3.4
L6	Daehan	29.1 ± 2.5
L7	Seomyeongsa 4	28.2 ± 2.7
L8	Gongju 1	28.1 ± 3.3
L9	Mipung	27.3 ± 2.6
L10	Jinju 2	27.3 ± 2.8
L11	Seomyeongsa	27.2 ± 2.3
L12	Dongnong 3	27.0 ± 2.1
L13	Soposi 2	27.0 ± 2.1
L14	Seomyeongsa 2	26.8 ± 2.3
L15	Gyeongje	26.7 ± 2.4
L16	Gwangdeok	26.7 ± 3.0
L17	Jeongwol	26.7 ± 2.1
L18	Joyul	26.6 ± 3.0
L19	Deokmyeong	26.5 ± 2.4
L20	Janggwangsa	25.7 ± 1.6
L21	Burim	25.2 ± 3.7
S1	Maepyeongjosaeng	14.2 ± 2.3
S2	Kurakataamaguri	13.5 ± 1.9
S3	Kwangjujoyul	13.2 ± 1.7
S4	Pungeun	13.1 ± 1.4
S5	Gwangju 2	13.0 ± 1.4
S6	Daejeonyul	12.8 ± 2.1
S7	Ilyachun	12.8 ± 2.0
S8	Gwangju 3	12.6 ± 1.7
S9	Jinju 1	11.8 ± 2.2
S10	Toyotamawase	11.3 ± 2.5
S11	Ogbijosaeng	11.0 ± 1.7
S12	Ibwon 1	10.7 ± 1.5
S13	Bukgwan	10.6 ± 1.6
S14	Bangsa	10.3 ± 1.4
S15	Osibpa	8.9 ± 1.5
S16	Sangmyeon 1	8.8 ± 1.7
S17	Panyul	7.0 ± 1.6
S18	Amsu 3	8.9 ± 1.7
S19	Sohwagwang	5.4 ± 1.1
S20	Jangwon	8.8 ± 2.0
S21	Hamjongyul	4.8 ± 0.8

Supplementary Table S2. List of chestnut accessions for the validation sets (n=46).

No.	Name	Nut weight (g/FW)
VP1	Jeongseon 13	7.5 ± 1.7
VP2	Gurye 15	7.8 ± 1.7
VP3	Hadong 13	8.1 ± 1.2
VP4	Hongchun 18	9.2 ± 2.3
VP5	Yanggu 8	10.0 ± 2.6
VP6	Gurye 1	10.1 ± 1.6
VP7	Macheon 2	10.1 ± 1.2
VP8	Hongchun 23	10.3 ± 1.7
VP9	Gurye 9	10.4 ± 1.8
VP10	Hadong 9	10.4 ± 1.1
VP11	Gangneung 7	10.6 ± 2.4
VP12	Jeongseon 11	10.8 ± 1.5
VP13	Gwacheon 1	10.9 ± 2.2
VP14	Uljin 2	10.9 ± 1.6
VP15	Gurye 8	11.0 ± 1.8
VP16	Uljin 10	11.1 ± 2.6
VP17	Uljin 12	11.7 ± 2.3
VP18	Jeongseon 10	12.2 ± 2.1
VP19	Hongchun 16	12.3 ± 1.8
VP20	Uljin 11	13.0 ± 2.7
VP21	Gangneung 4	13.1 ± 2.4
VP22	Hyeonri 1	13.7 ± 1.9
VP23	Hongchun 22	14.2 ± 1.9
VP24	Parkmi1	14.8 ± 2.0
VP25	Gwacheon 5	15.1 ± 2.4
VP26	Juok	16.4 ± 2.1
VP27	Akok	17.0 ± 3.3
VP28	Parkmi2	18.6 ± 2.7
VP29	Arima	19.1 ± 1.6
VP30	Daekukjosaeng	19.2 ± 2.5
VP31	Pyeonggi	19.2 ± 1.6
VP32	Hwacheon 12	19.7 ± 2.4
VP33	Ishizuchi	19.9 ± 2.3
VP34	Kwangeun	20.1 ± 2.3
VP35	Saeil	20.1 ± 2.2
VP36	Kunumi	20.5 ± 1.8
VP37	Daebo	20.5 ± 3.0
VP38	Riheiguri	20.9 ± 2.3
VP39	Sinyipyeong	21.3 ± 2.6
VP40	Euljong	21.3 ± 1.4
VP41	Sanyayul	22.0 ± 2.1
VP42	Daesan	22.3 ± 1.8
VP43	Sinmyeong	22.5 ± 2.2
VP44	Isseumo	22.7 ± 1.4
VP45	Mansung	24.5 ± 1.5
VP46	Sansung	25.0 ± 2.1

Supplementary Table S3. Statistics for RNA sequencing reads of 42 chestnut accessions.

No.	Raw reads	Clean reads	Mapped reads	Mapped rates, %	Uniquely Mapped	Uniquely Mapped rates, %
L1	48,841,916	47,663,124	40,650,685	85.3%	40,084,476	84.1%
L2	49,310,972	48,167,224	40,275,367	83.6%	39,555,842	82.1%
L3	47,616,934	46,520,440	38,967,889	83.8%	38,361,701	82.5%
L4	53,272,746	52,104,368	43,233,170	83.0%	42,596,760	81.8%
L5	55,760,734	54,517,952	44,280,886	81.2%	43,461,181	79.7%
L6	51,846,260	50,621,734	42,120,320	83.2%	41,536,554	82.1%
L7	51,698,758	50,513,032	41,186,844	81.5%	40,579,554	80.3%
L8	49,651,938	48,507,026	39,919,039	82.3%	39,319,743	81.1%
L9	51,543,716	50,452,886	42,936,826	85.1%	42,245,341	83.7%
L10	50,752,022	49,735,726	42,535,223	85.5%	41,917,283	84.3%
L11	53,782,130	52,629,460	45,283,822	86.0%	44,601,416	84.7%
L12	48,325,820	47,417,790	40,507,523	85.4%	39,924,109	84.2%
L13	53,262,948	52,197,208	44,598,191	85.4%	43,941,952	84.2%
L14	53,482,532	52,490,370	44,709,100	85.2%	44,042,479	83.9%
L15	49,556,680	48,509,878	41,364,052	85.3%	40,722,520	83.9%
L16	45,626,906	44,667,782	37,977,722	85.0%	37,340,660	83.6%
L17	49,331,210	48,420,698	41,498,833	85.7%	40,858,456	84.4%
L18	49,708,030	48,785,850	41,650,082	85.4%	41,038,262	84.1%
L19	64,199,482	62,715,698	51,390,338	81.9%	50,576,799	80.6%
L20	64,379,290	63,005,606	51,606,777	81.9%	50,788,884	80.6%
L21	55,103,178	53,868,564	44,577,771	82.8%	43,937,699	81.6%
S1	68,272,514	66,545,142	54,555,186	82.0%	53,782,844	80.8%
S2	49,780,434	48,791,820	42,449,449	87.0%	41,843,291	85.8%
S3	49,258,168	48,351,102	40,854,717	84.5%	40,298,809	83.3%
S4	46,068,500	44,982,314	37,771,134	84.0%	37,158,974	82.6%
S5	47,085,510	45,914,510	38,744,537	84.4%	38,146,570	83.1%
S6	49,725,380	48,575,056	41,613,207	85.7%	40,876,955	84.2%
S7	47,323,602	46,299,622	41,226,730	89.0%	40,616,896	87.7%
S8	56,555,836	55,277,040	45,861,031	83.0%	45,180,798	81.7%
S9	47,267,300	46,069,434	39,199,357	85.1%	38,597,507	83.8%
S10	52,922,200	51,497,478	42,704,883	82.9%	42,091,926	81.7%
S11	49,442,904	48,173,102	40,967,703	85.0%	40,332,933	83.7%
S12	52,740,360	51,154,260	42,257,023	82.6%	41,526,099	81.2%
S13	58,180,294	56,665,716	47,620,763	84.0%	46,748,217	82.5%
S14	53,615,946	52,018,984	45,645,455	87.7%	44,948,724	86.4%
S15	55,588,454	53,901,256	45,681,894	84.8%	44,844,423	83.2%
S16	60,970,642	59,170,680	50,940,847	86.1%	50,063,928	84.6%
S17	58,491,110	56,859,432	47,875,365	84.2%	46,873,694	82.4%
S18	47,219,808	45,899,368	37,788,767	82.3%	37,245,371	81.1%
S19	52,675,528	51,155,564	42,758,934	83.6%	42,122,563	82.3%
S20	46,664,890	45,460,286	38,148,392	83.9%	37,625,401	82.8%
S21	51,286,982	50,009,964	43,775,974	87.5%	43,093,135	86.2%
**Total**	**2,198,190,564**	**2,146,284,546**	**1,809,711,808**		**1,781,450,729**	
**Average**	**52,337,871**	**51,102,013**	**43,088,376**	**84.4%**	**42,415,494**	**83.1%**

Supplementary Table S4. Distribution of variant numbers in different types.

Types	Count	Percent
EXON	217,133	33.5%
INTRON	23,391	3.6%
UTRs	407,865	62.9%

Supplementary Table S5. Performance of machine learning algorithms in training (n=42) and validation datasets (n=46). AUC, area under the ROC curve, SVM, support vector machines, KNN, k-nearest neural network, RF, random forest, PLS, partial least squares.

Algorithm	Training sets (n=42)			Validation sets (n=46)		
	AUC	Sensitivity	Specificity	AUC	Sensitivity	Specificity
SVM	1	0.95	1	0.62	0.03	0.94
KNN	1	0.95	1	0.80	0.45	0.84
RF	1	0.96	1	0.80	0.55	0.81
C5.0	0.94	0.98	0.92	0.75	0.42	0.86
PLS	1	0.95	1	0.78	0.53	0.86

Supplementary Table S6. Performance of machine learning algorithms with SNPs from Set A to H in training datasets (n=42). AUC, Area under the ROC curve, SVM, support vector machines, KNN, k-nearest neural network, RF, random forest, PLS, partial least squares.

Marker selection	Set	FDR	Algorithm	AUC	Sensitivity	Specificity
Categorical mode	Set A	0.05	C5.0	0.90	0.91	0.88
			KNN	1	1	1
			PLS	1	1	1
			RF	1	1	1
			SVM	1	0.99	1
	Set B	0.01	C5.0	0.90	0.91	0.89
			KNN	1	1	1
			PLS	1	1	1
			RF	1	1	1
			SVM	1	0.99	1
	Set C	0.005	C5.0	0.92	0.92	0.90
			KNN	1	1	1
			PLS	1	1	1
			RF	1	1	1
			SVM	1	1	1
	Set D	0.001	C5.0	0.96	0.96	0.95
			KNN	0.98	1	0.96
			PLS	1	1	0.95
			RF	1	1	0.96
			SVM	1	0.99	0.94
Quantitative mode	Set E	0.05	C5.0	0.91	0.91	0.90
			KNN	1	1	1
			PLS	1	1	1
			RF	1	1	1
			SVM	1	1	1
	Set F	0.01	C5.0	0.91	0.91	0.90
			KNN	1	1	1
			PLS	1	1	1
			RF	1	1	1
			SVM	1	1	0.99
	Set G	0.005	C5.0	0.92	0.94	0.90
			KNN	1	1	1
			PLS	1	1	1
			RF	1	1	0.99
			SVM	1	1	0.99
	Set H	0.001	C5.0	0.93	0.95	0.90
			KNN	1	1	1
			PLS	1	1	1
			RF	1	1	1
			SVM	1	1	1

Supplementary Table S7. List of primer sequences used in Sanger sequencing.

Contig ID (Scaffold No : SNP Location)	Forward (5'-3')	Reverse (5'-3')
scaffold01190:13428	TCTGGGCATGAACCATTCTACC	TCGGATAGGGACTTGATGGTCT
scaffold01019:66232	TTTTTGGTGTAGGGTTTGCTGG	TACGACACCTCTTGAATTGGGG
scaffold01019:66350	TTTTTGGTGTAGGGTTTGCTGG	TACGACACCTCTTGAATTGGGG
scaffold00551:8613	GCCCTCTTAATTGAGCCCTCTC	TGTGAGTGTGCTGAACCTGAAT
scaffold00859:85468	TGTTCCGATGGCTTGAAAACTG	CTCATGTGTGCAATGTGATCCC
scaffold00485:18530	AGTGGGTCTTTGAAGAGGATGC	TGCATCACAGGTATGGAGCAAT
scaffold00406:56142	ACTGTGAACTCTGATTGGCTGT	CTTCATGTACCCCCAGGACAAG
scaffold00485:49474	AGTGGTCATGGGAGCTTCTCTA	GGCCTTCATCCTATGATTGACAC
scaffold01190:53971	TCCAATGTTAGAACTGGCAGCT	CGGGGTCGAATTTTACTTGTGG
scaffold00406:46801	TGTCTATGAACAGTGATGTGATGCT	GTGCACTGAGTTTCTCTTGTGA
scaffold00406:46909	TGTCTATGAACAGTGATGTGATGCT	TTATCTTGTAAGGTATCATCTG
scaffold00485:2380	TCCTGTAGGCTGTCAGTCTCTTC	ACACACAACACCAAAATTATACGG
scaffold00491:55296	CCTCTGGCACTTCAATGAGGAT	TGATGGTGTCGAAGTGAGAAGG
scaffold00859:92916	TGGGTCTTGTTGCTTTGGGTAT	GCAGTTCCACGATTTTGTTCCT
scaffold01019:57173	GTATCTGAAGGGAATCTGGATGG	GGATCAGCTCCCGCAAATAGTA
scaffold01019:57473	GTATCTGAAGGGAATCTGGATGG	GGATCAGCTCCCGCAAATAGTA
scaffold01019:66111	TTTTTGGTGTAGGGTTTGCTGG	TACGACACCTCTTGAATTGGGG
scaffold01190:53970	TCCAATGTTAGAACTGGCAGCT	CGGGGTCGAATTTTACTTGTGG
scaffold00485:53410	CCAATGGTTCCAAAAAGAGACT	CAGTGGCAAAGGATTCAACTGG
scaffold00551:85919	ATTTAAGAAGTGGCGGATGGGT	GCTTCAGGCCCTTTGGTTAATC
scaffold00551:98229	GAGTTGCATCCCACGTTTTTCA	GAAGGAAAACAGAGTGGTGTGC

Supplementary Table S8. Genotyping by Sanger sequencing with 21 SNPs on 46 validation datasets.

Major/ minor	G/T	A/G	G/A	G/A	C/T	C/T	C/T	T/C	G/A	C/T	G/C	A/G	T/A	A/T	A/G	G/A	T/C	G/A	T/C	C/T	A/G
ID / No.	13428	66232	66350	8613	85468	18530	56142	49474	53971	46801	46909	2380	55296	92916	57173	57473	66111	53970	53410	85919	98229
VP1	G	A	G	G	C	C	C	T	G	C/T	G	A	T	A	A	G	T	G	T	C	A
VP2	G	A	G	G	C	C	C	T	G	C	G	A	T	A	A	G	T	G	T	C	A
VP3	G	A	G/A	G	C	C	C	T	G	C	G	A	T	A	A	G	T	G	T	C	A
VP4	G	A	G	G	C	C	C	T	G	C	G	A	T	A	A	G	T	G	T	C	A
VP5	G	A	G	G	C	C	C/T	T	G	C/T	G/C	G	T	A	A	G	T	G	T	C	A
VP6	T/G	A	G	G/A	C	C/T	C/T	T/C	G/A	C/T	G/C	A/G	A/T	A/T	A	G	T	G/A	T/C	C/T	A/G
VP7	T/G	A	G	G/A	C/T	C	C/T	T	G/A	C/T	G	A	T	A	A/G	G/A	T	G/A	T	C	A
VP8	G	A	G	G	C	C	C	T	G	C	G	A	T	A	A	G	T	G	T	C	A
VP9	G	A	G	G	C	C/T	C	T	G	C	G/C	A	T	A	A	G	T	G	T	C	A
VP10	G	A	G	G	C	C	C	T	G	C	G	A	T	A	A	G	T	G	T	C	A
VP11	G	A/G	G/A	G/A	C	C	C	T/C	G	C	G	A	T	A	A	G	C/T	G	T/C	C	A
VP12	G	A	G	G	C	C	C	T	G	C	G	A	T	A	A	G	T	G	T	C	A/G
VP13	G	A/G	G/A	G	C	C	C/T	T	G	C	G	A	T	A	A/G	G/A	C/T	G	T	C	A
VP14	G	A	G	G	C	C	C	T	G	C	G	A	T	A	A/G	G	T	G	T	C	A
VP15	G	A	G	G	C	C	C	T	G	C	G	A	T	A	A	G	T	G	T	C	A
VP16	G	A	G	G	C	C	C	T	G	C	G	A	T	A	A	G	T	G	T	C	A
VP17	G	A	G	G	C	C/T	C	T	G	C	G/C	A	T	A	A	G	T	G	T	C	A
VP18	T/G	A/G	G/A	G	C	C/T	C	T/C	G/A	C/T	G/C	A	T	A	A	G/A	C/T	G/A	T	C/T	A
VP19	G	A	G	G	C	C	C	T	G	C	G/C	A	T	A	A	G	T	G	T	C	A
VP20	G	A/G	G/A	G	C	C	C	T	G	C	G/C	A	T	A	A/G	G/A	C/T	G	T	C	A
VP21	G	A	G	G	C	C	C	T	G	C/T	G	A	T	A	A	G	T	G	T	C	A
VP22	G	A	G	G	C	C	C	T	G	C	G	A	T	A	A	G	T	G	T	C	A
VP23	G	A	G	G	C	C	C	T	G	C	G	A	T	A	A	G	T	G	T	C	A
VP24	G	A	G	G	C	C	C	T	G	C	G/C	A	T	A	A	G	T	G	T	C	A
VP25	G	A	G	G	C	C	C	T	G	C/T	G	A	T	A	A	G/A	T	G	T	C	A
VP26	T/G	A/G	G/A	G/A	C	C	C	T	G/A	C	G	A	T	A	A/G	G/A	C/T	G/A	T	C/T	A/G
VP27	G	A	G	G	C	C	C	T	G	C	G	A	T	A/T	A	G	T	G	T	C	A
VP28	T/G	A	G	G/A	C/T	C/T	C	T/C	G/A	C	G/C	A/G	A/T	A/T	A	G	T	G/A	T/C	C/T	A/G
VP29	T/G	A/G	G/A	G/A	C/T	C/T	C/T	T/C	G/A	T	G/C	A/G	A/T	A/T	A/G	G/A	C/T	G/A	T/C	C/T	A/G
VP30	G	A/G	G/A	G	C	C	C/T	T/C	G	C/T	G	A	T	A	A/G	G/A	C/T	G	T	C	A
VP31	G	A	G	G/A	C	C	C/T	T/C	G	C/T	G/C	A	T	A	A	G/A	T	G	T/C	C	A
VP32	T	G	A	A	C/T	C/T	C/T	T/C	A	C/T	G/C	A/G	A/T	A/T	G	A	C	A	T/C	T	A/G
VP33	T/G	A	G	G/A	C/T	C/T	C/T	T/C	G/A	C/T	G/C	G	A	A/T	A	G	T	G/A	T/C	C/T	A/G
VP34	G	A/G	G/A	G	C	C	C	T	G	C	G	A/G	A/T	A/T	A	G	C/T	G	C	C/T	A/G
VP35	G	A	G	G	C	C	C	T	G	C	G	A/G	T	A	G	G	T	G	T	C/T	A
VP36	T/G	A	G	G/A	C	C	C	T/C	G	C/T	G	A/G	A	A/T	A	G	T	G	T/C	C/T	A/G
VP37	T	A/G	G/A	G/A	C/T	C/T	T	T/C	A	C/T	G/C	A/G	A/T	A/T	A/G	G/A	C/T	A	T/C	C/T	A
VP38	T/G	A	G	G/A	C/T	C/T	C/T	T/C	G/A	C/T	G/C	A/G	A/T	A/T	A	G	T	G/A	T/C	C/T	A/G
VP39	G	A	G/A	A	C	C	C	T	G	C	G	A	T	A	A	G	T	G	T	C	A/G
VP40	T/G	A/G	G/A	G/A	C/T	C/T	C/T	T/C	G/A	C/T	G/C	A/G	A/T	A/T	A/G	G/A	C/T	G/A	T/C	C/T	A/G
VP41	T/G	A	G	G/A	C	C/T	C	T/C	G/A	C	G/C	A/G	A/T	A/T	A	G	T	G/A	T/C	C/T	A/G
VP42	T	A/G	G/A	A	C/T	T	T	C	A	T	C	G	A/T	A/T	A/G	G/A	C/T	A	C	C/T	G
VP43	T	A	G	A	C/T	T	C/T	T/C	A	C/T	C	A	A/T	A/T	A/G	G/A	T	A	T/C	T	G
VP44	T/G	A	G	G/A	C/T	C/T	C/T	C	G/A	C	G/C	A/G	A/T	T	A	G	T	G/A	T	C/T	G
VP45	T/G	A/G	G/A	G/A	C/T	C/T	C/T	T/C	G/A	C/T	G/C	A/G	T	A/T	A/G	G/A	C/T	G/A	T/C	C/T	A/G
VP46	G	A	G	G/A	C	C	C	T	G	C/T	G	A	T	A	A	G	T	G	T	C	A
